# Dielectric properties and lamellarity of single liposomes measured by in-liquid scanning dielectric microscopy

**DOI:** 10.1186/s12951-021-00912-6

**Published:** 2021-06-03

**Authors:** Martina Di Muzio, Ruben Millan-Solsona, Aurora Dols-Perez, Jordi H. Borrell, Laura Fumagalli, Gabriel Gomila

**Affiliations:** 1 Institut de Bioenginyeria de Catalunya (IBEC), The Barcelona Institute of Science and Technology (BIST), c/Baldiri i Reixac 11-15, 08028 Barcelona, Spain; 2 Departament D’Enginyeria Electrònica I Biomèdica, Universitat de Barcelona, C/Martí i Franqués 1, 08028 Barcelona, Spain; 3 Secció de Fisicoquímica, Facultat de Farmàcia I Ciències de L’Alimentació, Universitat de Barcelona, Av. Diagonal, 643, 08028 Barcelona, Spain; 4 Department of Physics and Astronomy, University of Manchester, Manchester, M13 9PL UK; 5 National Graphene Institute, University of Manchester, Manchester, M13 9PL UK

**Keywords:** Liposomes, Lamellarity, Membrane capacitance, Scanning dielectric microscopy, Nanoscale

## Abstract

Liposomes are widely used as drug delivery carriers and as cell model systems. Here, we measure the dielectric properties of individual liposomes adsorbed on a metal electrode by in-liquid scanning dielectric microscopy in force detection mode. From the measurements the lamellarity of the liposomes, the separation between the lamellae and the specific capacitance of the lipid bilayer can be obtained. As application we considered the case of non-extruded DOPC liposomes with radii in the range ~ 100–800 nm. Uni-, bi- and tri-lamellar liposomes have been identified, with the largest population corresponding to bi-lamellar liposomes. The interlamellar separation in the bi-lamellar liposomes is found to be below ~ 10 nm in most instances. The specific capacitance of the DOPC lipid bilayer is found to be ~ 0.75 µF/cm^2^ in excellent agreement with the value determined on solid supported planar lipid bilayers. The lamellarity of the DOPC liposomes shows the usual correlation with the liposome's size. No correlation is found, instead, with the shape of the adsorbed liposomes. The proposed approach offers a powerful label-free and non-invasive method to determine the lamellarity and dielectric properties of single liposomes.

**Supplementary Information:**

The online version contains supplementary material available at 10.1186/s12951-021-00912-6.

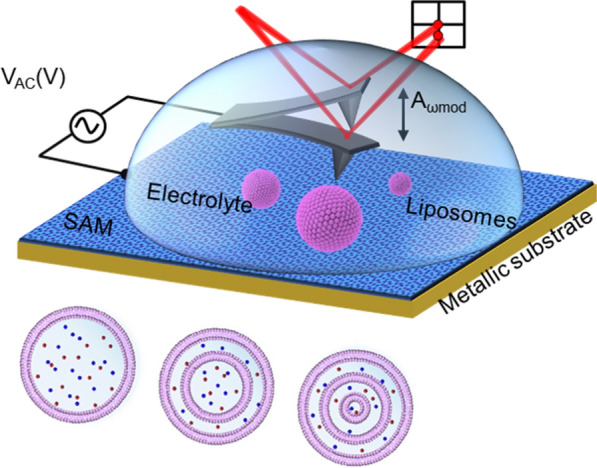

## Background

Liposomes consist of single or multiple concentric lipid bilayers encapsulating an aqueous compartment. Their sizes range from tens of nanometres to tens of micrometres [1]. Liposomes are widely used as drug delivery nanocarriers [2–4] and as cell model systems for the study of cell membrane properties [5], transmembrane processes and intracellular biochemistry [6].

Several techniques exist to probe the structural, physical and chemical properties of liposomes [7–9]. Among the structural properties, lamellarity, i.e. the number of concentric lipid bilayers in a liposome, is among the most relevant ones. Lamellarity, determines the stability of liposomes' preparations [10], the amount of lipophilic drugs that can be encapsulated, the kinetics of their release [4] and the fate of liposomes when interacting with cells [7]. Moreover, it also determines the overall mechanical [11] and dielectric [12] properties of the liposomes, which are relevant in their interaction with cells [13, 14] or in their electrokinetic manipulation [15]. Finally, when used as cell model systems, strict control of the lamellarity is a pre-requisite [6].

Lamellarity can be determined at the population level by using techniques such as Nuclear Magnetic Resonance, X-ray small angle scattering and fluorescence spectroscopy [16, 17]. These techniques provide the average lamellarity of the liposomes' population. Lamellarity can be determined, also, at the single liposome level by considering imaging techniques such as cryo-electron microscopy, freeze-fracture electron microscopy and light microscopy [18, 19], or mechanical techniques such as micropipette aspiration [20, 21], and more recently, atomic force microscopy (AFM) and force spectroscopy measurements [11]. Despite their success, the existing techniques to determine the lamellarity of single liposomes present the main drawback of either being invasive/destructive or making use of labels and staining agents. Developing techniques that are simultaneously non-invasive and label-free would be desirable for some applications, as for instance, in the in-situ analysis of the variation of lamellarity due to a change of the environmental conditions, the time elapsed upon adsorption, or its interaction with molecules and biomolecules.

A possible route to determine the lamellarity of single liposomes in a non-invasive and label-free way is to consider the dielectric response of liposomes in external ac electric fields. This route has been explored in electrorotation measurements [12, 22], in which the rotation velocity of a liposome suspended in a rotating electric field cage is determined as a function of the frequency of the external ac potential. From these measurements the lamellarity of the liposome can be inferred since the electric force is long ranged and, hence, probes the liposomes' interior [12, 22]. However, downscaling electrorotation measurements to probe sub-micrometric liposomes is very challenging and has not been achieved, yet. An alternative approach could consist in the use of nanoscale dielectric scanning probe microscopy techniques. Quantitative dielectric measurements on nanoscale objects down to 10 nm resolution was demonstrated using the approach that we referred to as Scanning Dielectric Microcopy (SDM), in which the capacitive interaction between a sharp probe and the small-scale object is quantified using analytical or numerical models either in air or in liquid [23–26]. Using this approach, subsurface dielectric information has been obtained on various micro and nano structures, including single viruses [25, 27], bacterial cells [28], endospores [29], and, recently, water-filled buried nanochannels [30]. In particular, by using SDM in air [25, 27], DNA-filled viral capsids as small as 60 nm in diameter could be discriminated from empty viral capsids, and the dielectric polarization properties of the protein shell and of the DNA contained inside could be determined in a non-invasive manner and without labels. In the context of material sciences, dielectric scanning probe microscopy techniques have been used to probe the presence of buried carbon nanotubes in polymer matrices [31–37].

In the present work we demonstrate that the lamellarity of single liposomes can be determined in a non-invasive and label-free way by using scanning dielectric microscopy in force detection mode operated in liquid environment [26]. In-liquid SDM in force detection mode overcomes some of the limitations of SDM in current sensing mode [38, 39] concerning spatial resolution and sensitivity. It has been used until now to determine the local specific capacitance of thin dielectric films [26], solid supported lipid bilayers [40, 41] and nanopatterned self-assembled monolayers [42], and the local conductivity and interfacial capacitance of electrolyte gated field effect transistors [43]. Here, we show that it can also be used to probe the internal structure of single liposomes in electrolyte solutions. The proposed approach also provides the specific capacitance of the lipid bilayers forming the liposomes, a key parameter in Bioelectricity [44].

## Results

**Fig. 1:**
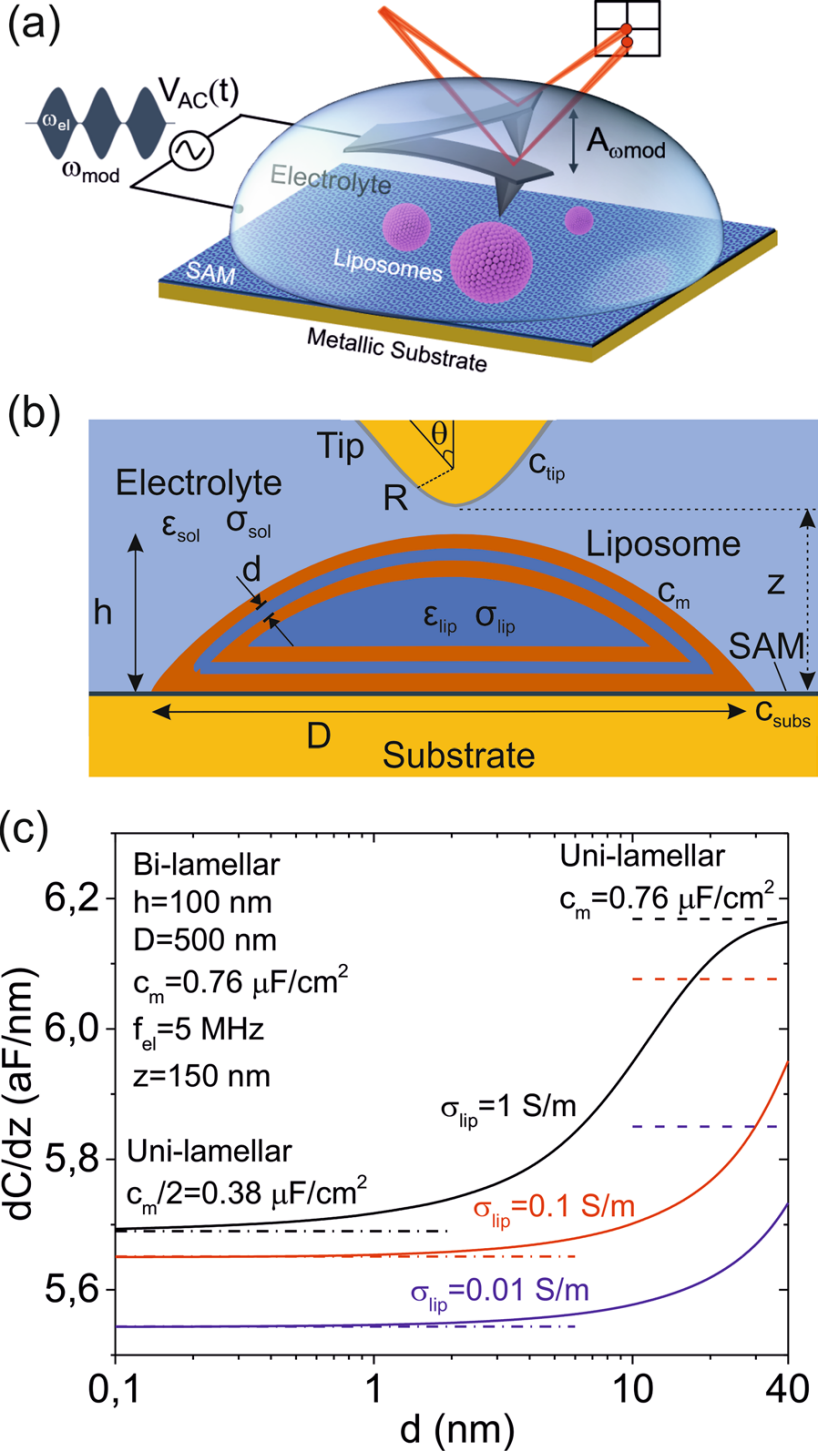
**a** Schematic representation of the in-liquid SDM setup in force detection mode used to measure the dielectric properties of single liposomes adsorbed on a functionalized metallic substrate. **b** Schematic representation of the theoretical model used to compute the tip-liposome capacitance gradient, *dC/dz*, for the case of an adsorbed bi-lamellar liposome, with the different parameters indicated. Not included in the scheme: tip height *H*, disc cantilever length *L*_*c*_ and cantilever thickness, *W*. **c** (continuous lines) Numerically calculated dependence of the tip-liposome capacitance gradient, *dC/dz*, for a bi-lamellar liposome as a function of the separation between the lamellae, *d*, for three different conductivities of the internal solution, σ_*lip*_ = 0.01 S/m, 0.1 S/m, 1 S/m. The dashed and dot-dashed lines correspond to the capacitance gradient values, *dC/dz*, for a uni-lamellar model with membrane capacitances *c*_*m*_ = 0.76 µF/cm^2^ and *c*_*m*_/2 = 0.38 µF/cm^2^, respectively. Parameters of the calculations: tip radius, *R* = 30 nm, half cone angle, *θ* = 20°, cone height, *H* = 12.5 µm, cantilever disc radius, *L*_*c*_ = 3 µm, cantilever disc thickness, *W* = 3 µm, tip-substrate distance, z = 150 nm, spherical cap liposome shape with height, *h* = 100 nm and diameter, *D* = 500 nm, internal and external solution dielectric constants, *ε*_*sol*_ = *ε*_*lip*_ = 78, external solution conductivity, σ_*sol*_ = 0.2 µS/m, tip interfacial capacitance, *c*_*tip*_ = 2.7 µF/cm^2^, substrate interfacial capacitance, *c*_*subs*_ = 0.65 µF/cm^2^, frequency of the applied voltage, *f*_*el*_ = 5 MHz

The methodology used here to measure the dielectric properties of single liposomes is the one used earlier to probe the dielectric properties of single dielectric core–shell nano-objects such as viruses in air environment [25, 27], here adapted to work in an electrolyte solution [26, 42]. A schematic representation of the set-up is shown in Fig. 1a. Briefly, a commercial metallic AFM probe with tip radius in the range ~ 10–100 nm scans the sample in a two-pass mode. In the first pass, the topography of the sample is recorded in intermittent contact mode. In the second pass the electric polarization of the liposomes, represented by the capacitance gradient, *dC/dz*, is recorded by measuring the force acting on the tip while scanning it at a constant height with respect to the substrate with a high frequency (*f*_*el*_ ~ 10 MHz) ac voltage applied. For a better signal to noise ratio the amplitude of the voltage is modulated at a low frequency (*f*_*mod*_ ~ kHz) [26]. The magnitude measured in in-liquid SDM is the oscillation amplitude of the cantilever at the modulation frequency, *A*_*ωmod*_, from where the ac electric force acting on the cantilever at this frequency, *F*_*ωmod*_, is obtained. The tip-liposome capacitance gradient *dC/dz* is then obtained from the relation *F*_*ωmod*_ = *1/8 dC/dz v*_*0*_^*2*^, where *v*_*0*_ is the amplitude of the ac applied voltage [26, 42, 45].

The tip-liposome capacitance gradient, *dC/dz*, contains information on the structural and dielectric properties of the liposome. Explicitly, the capacitance gradient, *dC/dz*, depends on the adsorbed liposome's size and geometry (determined by its height, *h*, and diameter, *D*), the liposome's internal structure (given by the number of lamellae, *n*, and the separation between them, *d*) and the specific capacitance of the lipid bilayer, *c*_*m*_, where *c*_*m*_ = *ε*_*0*_ *ε*_*m*_*/t*_*m*_, with *t*_*m*_ and *ε*_*m*_ being the thickness and dielectric constant of the lipid bilayer, respectively, and *ε*_*0*_ the vacuum permittivity. Moreover, *dC/dz* depends on the electrical properties of the internal and external solutions (given by the conductivities, σ_*lip*_ and σ_*sol*_, and the dielectric constants, *ε*_*lip*_ and *ε*_*sol*_, respectively), the tip-substrate distance, *z*, the frequency of the applied voltage, *f*_*el*_, the tip geometry (tip radius, *R*, half cone angle *θ*, tip height *H*, cantilever disc length, *L*_*c*_, and cantilever thickness, *W*), and the interfacial capacitances of the tip, *c*_*tip*_, and substrate, *c*_*subs*_ [40, 42] (see Fig. 1b).

Due to the non-trivial tip-liposome geometry we cannot use analytical expressions to determine accurately the tip-liposome capacitance gradient, *dC/dz*, and we must resort to numerical calculations to evaluate it (an approximate phenomenological relation is proposed in the discussion section). Figure 1c shows numerically calculated values of the tip-liposome capacitance gradient, *dC/dz*, as a function of the interlamellar separation for a bi-lamellar liposome of diameter *D* = 500 nm, height *h* = 100 nm and lipid bilayer specific capacitance *c*_*m*_ ~ 0.76 µF/cm^2^. The external aqueous solution is assumed to be of low conductivity (σ_*sol*_ = 0.2 µS/m, *ε*_*sol*_ = 78), adequate for in-liquid SDM measurements [45], while the internal one covers a range of values physiologically relevant, σ_*lip*_ = 0.01–1 S/m (*ε*_*lip*_ = 78). The tip radius, half cone angle and tip-substrate distance are *R* = 30 nm, *θ* = 20° and *z* = 150 nm, respectively, while the excitation frequency is *f*_*el*_ = 5 MHz, above the dielectric relaxation frequency of the external electrolyte solution, as required for in-liquid SDM measurements [45]. For comparison we also plotted the *dC/dz* values corresponding to uni-lamellar liposomes of the same size and shape with membrane specific capacitances *c*_*m*_ = 0.76 µF/cm^2^ and *c*_*m*_*/*2 = 0.38 µF/cm^2^ (dashed and dotted-dashed lines, respectively, in Fig. 1c). The tip-liposome capacitance gradient, *dC/dz*, (continuous lines in Fig. 1c) clearly depends on the interlamellar separation, *d*, demonstrating its sensitivity to the internal structure of the liposome. Its value evolves from the value corresponding to a uni-lamellar liposome with half the membrane specific capacitance, *c*_*m*_*/2*, for small interlamellar separations, to that of a uni-lamellar liposome with the given membrane specific capacitance, *c*_*m*_, for large interlamellar separations (the latter limit is reached only for the largest internal conductivities). This behaviour is consistent with the fact that at small interlamellar separations the liposome is like a uni-lamellar liposome with double membrane thickness and, hence, half specific capacitance, while for large interlamellar separations it behaves like a uni-lamellar liposome with the given membrane specific capacitance, since the internal lamella contributes little to the *dC/dz* value due to its very small surface area.

For liposomes with larger number of lamellae (n > 2) a similar behaviour is expected, with the capacitance gradient, *dC/dz*, at small inter-lamellar separations showing values corresponding to a uni-lamellar liposome with membrane specific capacitances *c*_*m*_*/*3, *c*_*m*_*/*4, etc. for tri-, tetra-, etc. lamellar liposomes, respectively. For large interlamellar separations *dC/dz* will tend to the value corresponding to a uni-lamellar liposome with specific capacitance *c*_*m*_. These results demonstrate that by measuring the tip-liposome capacitance gradient, *dC/dz*, with in-liquid SDM the lamellarity of single liposomes can be determined, as well as, the separation between lamellae and the specific capacitance of the lipid bilayer.

**Fig. 2:**
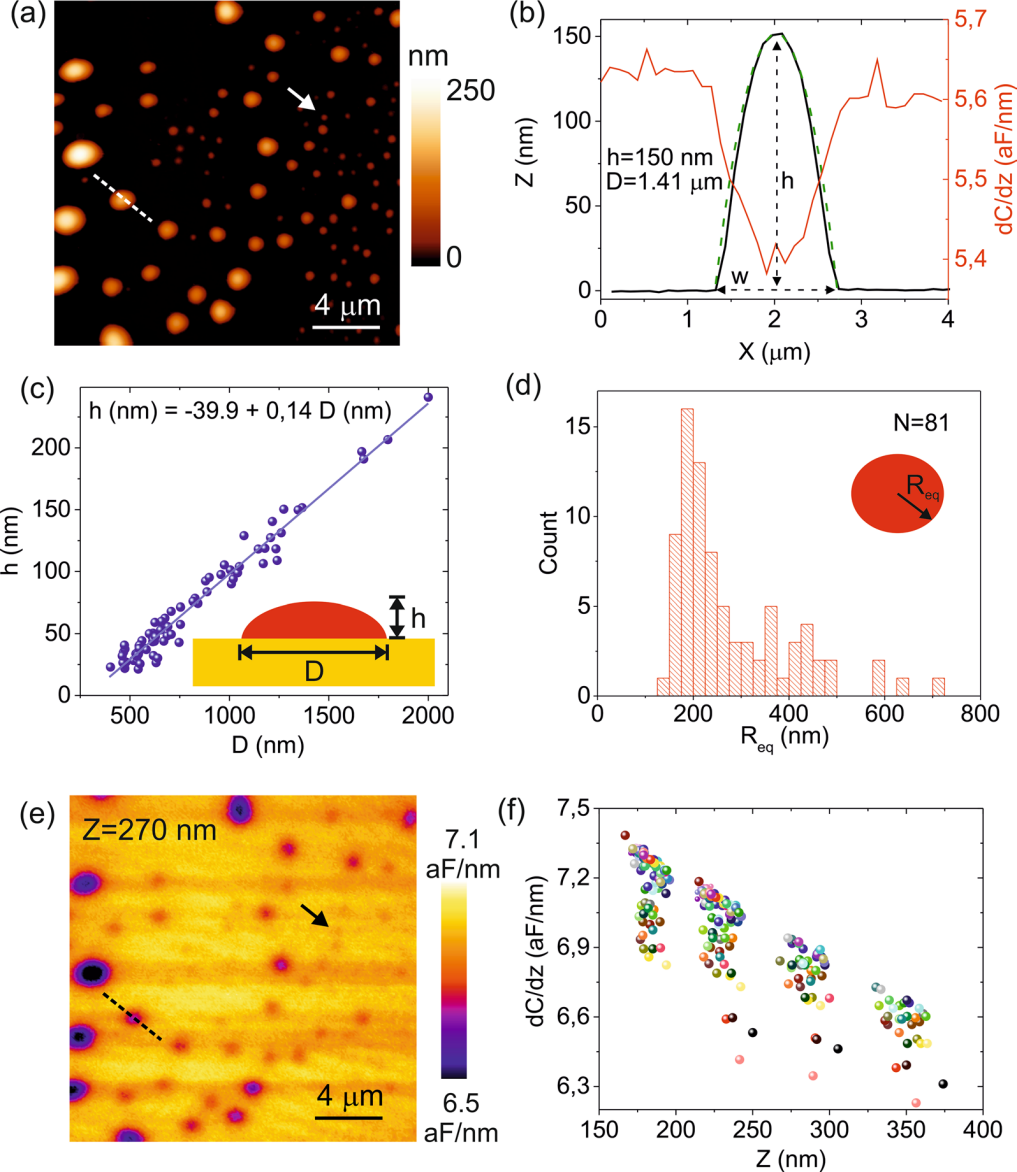
**a** AFM topographic image of non-extruded DOPC liposomes adsorbed on a functionalized planar gold substrate. **b** Cross-section topographic (black line) and capacitance gradient (red line) profiles along the dashed line in **a** and **e**, respectively. The green dashed line represents the adjustment of the profile to a spherical cap geometry. **c** (symbols) Height, h, versus width, D, of the liposomes in **a**. The continuous line is a linear fit to the data, which gives a slope (aspect ratio) ~ 0.14. **d** Distribution of equivalent spherical radii of the liposomes in **a** assuming that the surface area remains constant during the absorption process. **e** Constant height SDM image acquired at a distance *z* ~ 270 nm from the substrate and at a frequency *f*_*el*_ = 5 MHz. **f** Capacitance gradient at the centre of each detected liposome in the SDM images measured at four tip-substrate distances. Each colour represents the same liposome (*N* = 81). For the largest liposomes (*h* > 200 nm) data for *z* = 190 nm is not available. Experimental parameters: σ_*sol*_ = 0.2 µF/m, σ_*lip*_ = 0.8 S/m, *f*_*mod*_ = 6 kHz, *v*_*0*_ = 0.7 V, *k* = 0.43 N/m, *f*_*0*_ = 30 kHz (in air)

Figure 2a shows a topographic AFM image of non-extruded DOPC liposomes prepared by the hydration method adsorbed on a flat gold substrate functionalized with a Mercaptoethanol self-assembled monolayer. The liposomes have been prepared in a solution with moderate conductivity (σ_*lip*_ = 0.8 S/m) and imaged in a low conductivity solution (σ_*sol*_ = 0.2 µS/m) to facilitate the in-liquid SDM operation. The liposomes show an approximately spherical cap geometry (see topographic and spherical cap cross-section profiles, continuous black and dashed green lines in Fig. 2b). The heights of the adsorbed liposomes range from ~ 25 nm to ~ 250 nm and the diameters from ~ 250 nm to ~ 2 µm (see Fig. 1c). A linear relationship is found between the height and the diameter of the liposomes with a slope (aspect ratio) ~ 0.14. This very low aspect ratio implies a strong flattening of the DOPC liposomes when adsorbed on a substrate, what is consistent with the very soft nature of DOPC lipid bilayers at the temperature of the experiments (room temperature), where they are in the liquid phase [46]. From the measured heights and diameters, we have determined the distribution of equivalent spherical radii, *R*_*eq*_, by assuming the liposome's area remains constant during the adsorption process (Fig. 2d). The equivalent radii span the range of values ~ 100–800 nm. If one assumes, instead, that the volume remains constant the range of equivalent spherical radii would cover the range ~ 50–500 nm (see Additional file 1: S1).

Figure 2e shows a constant height in-liquid SDM image acquired at *f*_*el*_ = 5 MHz and at a tip substrate distance *z* = 270 nm, high enough to avoid touching any of the adsorbed liposomes.

The image shows a negative dielectric contrast for all the liposomes in the sample (see the capacitance gradient cross-section profile of one of the liposomes in Fig. 2b, red line). This fact implies that the equivalent homogeneous dielectric constant of the liposomes, *ε*_*eq*_, is smaller than that of the surrounding solution (*ε*_*sol*_ = 78) (see below).

The smallest liposome electrically detected in the SDM image in Fig. 2a has dimensions *h* = 23 nm and *D* = 402 nm, corresponding to an equivalent radius *R*_*eq*_ ~ 143 nm (marked with an arrow in Fig. 2a and e). SDM images at three additional tip-substrate distances (*z* = 175 nm, 215 nm and 325 nm) have been acquired (see Additional file 1: S2). Figure 2f shows the values of the capacitance gradient, *dC/dz*, at the centre of the liposomes (*N* = 81) and at four tip-substrate distances (for the largest liposomes no values at the closest distance are obtained, since the imaging distance is smaller than the height of those liposomes). Each colour represents one liposome. As expected, the capacitance gradient, *dC/dz*, decreases by increasing the imaging distance. In addition, for a given imaging distance, the values of *dC/dz* depend on the specific liposome considered, with the *dC/dz* values spanning a range of ~ 0.9 aF/nm, much larger than the measuring noise of the instrument ~ 0.03 aF/nm. This fact implies that different liposomes show different electric polarizations, what can be due to their different sizes, but also, due to their different internal structure, as shown below.

**Fig. 3:**
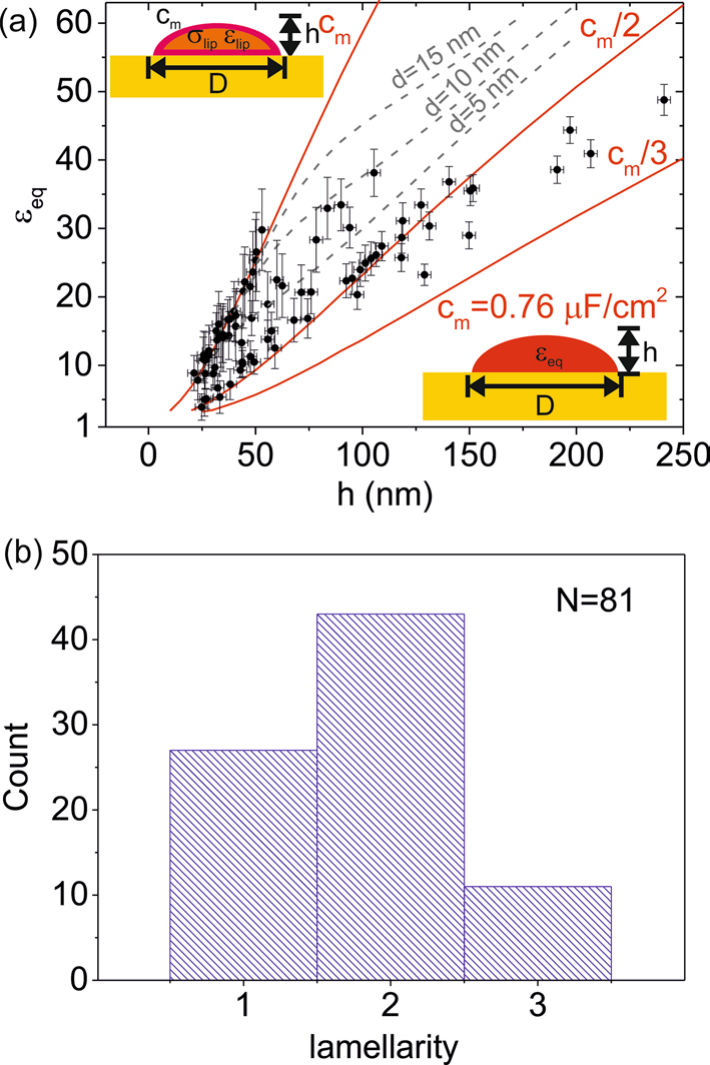
**a** (symbols) Equivalent homogeneous dielectric constant of the liposomes, *ε*_*eq*_, (*N* = 81) as a function of their height for the liposomes electrically detected in Fig. 2e. The vertical error bars correspond to the fitting error, while the horizontal ones to the uncertainty of the height determination. The model used in the calculations is shown in the bottom inset. The height and width have been determined from the topographic image in Fig. 2a. The tip geometry has been calibrated by using *dC/dz* approach curves acquired on a bare part of the substrate, giving *R* = 43 nm, *θ* = 20°, *C'*_*offset*_ = 1.5 aF/nm and *c*_*subs*_ = 0.6 µF/cm^2^. The remaining parameters of the tip and solution are the same as in Fig. 1c. Theoretical values for *ε*_*eq*_ predicted from the uni-lamellar liposome model shown in the top inset (red continuous lines), with membrane specific capacitances *c*_*m*_ = 0.78 µF/cm^2^, *c*_*m*_/2 and *c*_*m*_/3. For the internal solution we took σ_*lip*_ = 0.8 S/m, *ε*_*sol*_ = 78, corresponding to the solution used to prepare the liposomes. Theoretical values for *ε*_*eq*_ predicted for a bi-lamellar liposome model (grey dashed lines) with *c*_*m*_ = 0.78 µF/cm^2^ and interlamellar separations *d* = 5 nm, 10 nm and 15 nm. **b** Distribution of lamellarity values obtained from the analysis in **a** and corresponding to the liposomes electrically detected in Fig. 2e

To show that the measured *dC/dz* values are sensitive to the internal structure of the liposomes, we have determined the equivalent homogeneous dielectric constant, *ε*_*eq*_, of each liposome. *ε*_*eq*_ is the dielectric constant a homogeneous liposome should have in order to give the same capacitance gradient value, *dC/dz*, as the measured one [25]. If liposomes were homogeneous, *ε*_*eq*_ would be independent from the size and shape of the liposome. Instead, if some internal dielectric heterogeneity is present, *ε*_*eq*_ will depend on the size and shape of the liposomes (see Additional file 1: S3). To determine *ε*_*eq*_ we followed the methods of SDM [25], i.e. we calculated theoretical *dC/dz* vs distance curves for a homogeneous dielectric tip-liposome model (bottom inset in Fig. 3a) and fitted them to the experimental *dC/dz* vs distance values reported in Fig. 2f. Since the shape and size of each liposome are obtained from the topographic image, and the tip geometry and interfacial capacitances are calibrated from approach curves acquired on a bare part of the metallic substrate [25, 26, 42] (see Additional file 1: S4), *ε*_*eq*_ is the single fitting parameter (for the conductivity and dielectric constant of the external solution we took σ_sol_ = 0.2 µS/m and *ε*_sol_ = 78, respectively, although the actual conductivity of the solution does not play any role since the measuring frequency is above the relaxation frequency of the electrolyte). Figure 3a (symbols) shows the obtained values for the equivalent homogeneous dielectric constants *ε*_*eq*_ for the *N* = 81 liposomes electrically detected as a function of the height of each liposome. The equivalent homogeneous dielectric constant values, *ε*_*eq*_, span a range from ~ 4 to ~ 48, in all cases smaller than the dielectric constant of the external solution (*ε*_*sol*_ = 78), from where the negative contrast observed in the in-liquid SDM images (Fig. 2e), as anticipated before. The values of *ε*_*eq*_ show a clear dependence on the height of the liposomes, what is an unambiguous indication that the liposomes present some internal dielectric heterogeneity. As it is well known, the internal electrical heterogeneity of the liposomes comes from the presence of one (or several) lipid bilayers having very different electrical properties in comparison to those of the the enclosed solution. The dependence of *ε*_*eq*_ on the liposome height seem to display different "branches", rather than following a single dependence. This fact suggests that the internal dielectric heterogeneity of the different liposomes could be different.

To obtain further information on the internal structure of the liposomes, we have considered a uni-lamellar liposome model (top inset in Fig. 3a) with membrane specific capacitances *c*_*m*_, *c*_*m*_*/2* and *c*_*m*_*/3*. As we have shown above, this model predicts the behaviour expected for uni-, bi- and tri-lamellar liposomes when the interlamellar separation is very small. For the internal solution conductivity and dielectric constant, we used the values of the solution used to prepare the liposomes, σ_*lip*_ = 0.8 S/m and *ε*_*lip*_ = 78, respectively. For the specific capacitance of the lipid bilayer, *c*_*m*_, we took the value that matches the steepest dependence of *ε*_*eq*_ on the height. With this criterium, we obtained *c*_*m*_ = 0.78 µF/cm^2^, a value remarkably close to the one we obtained earlier with the same technique for solid supported planar DOPC bilayers (*c*_*DOPC*_ = 0.7–0.8 µF/cm^2^) [41]. The dependence of the equivalent homogeneous dielectric constant, *ε*_*eq*_, with the height of the liposomes predicted from the uni-lamellar models is shown by the continuous red lines in Fig. 3a. We observe that the theoretical curves for *c*_*m*_ and *c*_*m*_/2 nicely predict the trend of *ε*_*eq*_ with the height of many of the liposomes present in the sample. This result indicates that these *ε*_*eq*_ values correspond, respectively, to uni- and bi-lamellar liposomes, the latter having the lamellae close together. In addition, some values of *ε*_*eq*_ fall in between the curves corresponding to *c*_*m*_*/2* and *c*_*m*_*/3* and some others in between those corresponding to *c*_*m*_ and *c*_*m*_*/2*. According to the analysis presented above (Fig. 1b), these *ε*_*eq*_ values correspond to tri- and bi-lamellar liposomes, respectively, with the interlamellar separation, *d*, being relatively large. The interlamellar separation can be estimated for the case of bi-lamellar liposomes by considering the bi-lamellar model in Fig. 1b. Figure 3a (gray dashed lines) shows the dependence of *ε*_*eq*_ on the liposome height for bi-lamellar liposomes with interlamellar separations, *d* = 5 nm, 10 nm and 15 nm. The experimental values fall mostly around the d = 5 nm and d = 10 nm lines, indicating that the separation between the lamellae in the bi-lamellar liposomes is below ~ 10 nm. Performing a similar analysis for tri-lamellar liposomes involves too many unknowns and cannot provide unambiguous answers.

The previous analysis allows identifying the lamellarity of each individual liposome, and then building the distribution of lamellarities in the sample. Figure 3b shows the obtained lamellarity distribution in the case analyzed. Bi-lamellar liposomes are the more abundant ones (~ 53%), followed by uni-lamellar liposomes (~ 33%) and tri-lamellar liposomes (~ 14%). The lamellarity values reported here are minimum lamellarity values, since there can exist lamellae located deep inside the liposomes that are not detectable with the proposed technique (see below).

**Fig. 4:**
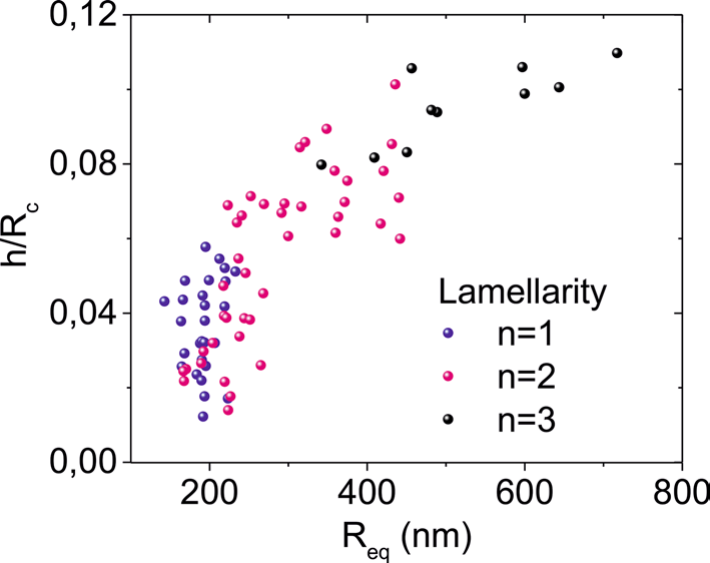
Geometrical shape ratio *h/R*_*c*_ as a function of the liposome equivalent spherical radius, *R*_*eq*_, for the liposomes electrically analyzed in Fig. 3. Symbols have been colored according to the number of lamellae

We highlight that with the proposed approach, like in other single liposome lamellarity detection techniques, one has access to also the size and shape of the liposomes. Therefore, one can investigate whether there is any relationship between the lamellarity and these parameters. For this purpose, we characterized the size and shape of the liposomes by considering the equivalent spherical radius, *R*_*eq*_, and the ratio *h/R*_*c*_ where *R*_*c*_ is the radius of curvature of the absorbed liposomes. Approximating their shape by a spherical cap, the radius of curvature is given by *R*_*c*_ = *[(D/2)*^*2*^ + *h*^*2*^*]/2 h*. The parameter *h/R*_*c*_ for flattened liposomes contains the same information than the contact angle, γ, since *h/Rc* = *1-cos(*γ*)* for γ < *90*. The ratio *h/R*_*c*_ has been used in earlier works for the analysis of the lamellarity of adsorbed liposomes by AFM force spectroscopy [11], while the contact angle γ has been used for the analysis of the stiffness of adsorbed liposomes [46]. Figure 4 shows the dependence of the ratio *h/R*_*c*_ on the equivalent spherical radius, *R*_*eq*_, for the liposomes analyzed in the present work. The color of the symbols refers to the number of lamellae as determined from the dielectric measurements (Fig. 3).

Figure 4 shows that some correlation exists between lamellarity and size, in agreement with the usual liposome classification methods [7]. In the present case, large liposomes (500 nm < *R*_*eq*_ < 800 nm) are mainly tri-lamellar; those of intermediate sizes (250 nm < *R*_*eq*_ < 500 nm) are mostly bi-lamellar; finally, small liposomes (*R*_*eq*_ < 250 nm) are either uni- or bi-lamellar. Large uni-lamellar liposomes (with *R*_*eq*_ > 250 nm) are not found in this sample. Concerning, the liposome's shape, represented by the parameter *h/R*_*c*_, it does not seem to provide additional information on lamellarity, besides the one already given by the size. This result could be surprising since lamellarity has been shown to affect (slightly) the stiffness of liposomes [11], and the stiffness has been, in turn, correlated with the liposome's shape (contact angle), once absorbed [46]. Therefore, it would be reasonable to expect that some relation would exist between the shape of adsorbed liposomes and their lamellarity. The lack of correlation for the case of DOPC liposomes could be due to their very soft nature, for which the relation between shape and stiffness could not be precisely established either [46]. The fact that in-liquid SDM measurements can determine the lamellarity of liquid-phase liposomes constitutes an added value of the proposed methodology with respect to mechanical methods.

**Fig. 5:**
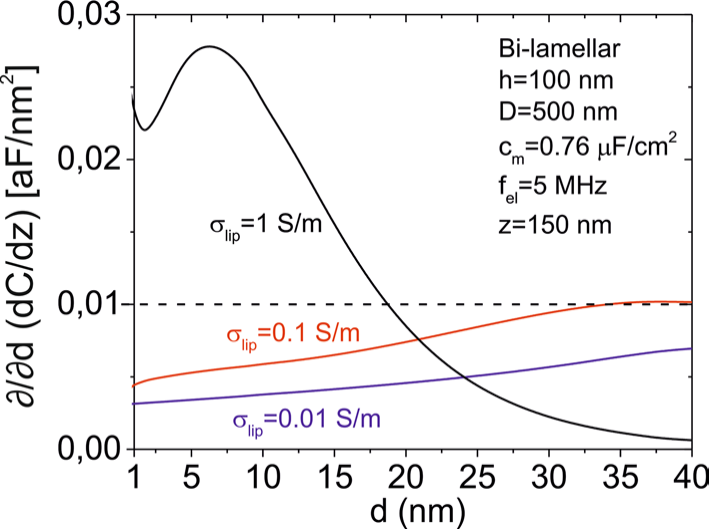
Sensitivity of *dC/dz* to the interlamellar separation, *d*, as a function of the interlamellar separation for a bi-lamellar liposome with height *h* = 100 nm and diameter *D* = 500 nm for three different internal conductivities, σ_*lip*_ = 0.01 S/m, 0.1 S/m and 1 S/m. The dashed line represents the sensitivity required to detect an interlamellar separation variation of 5 nm for an instrumental noise of 0.05 aF/nm. The parameters not specified are the same as those in Fig. 1c

In-liquid SDM measurements have shown a high sensitivity to the inter-lamellar separation, what enabled to estimate the interlamellar separation in the case of bi-lamellar liposomes. The range of interlamellar separations that are actually accessible by means of this approach is, in general limited. Figure 5 shows the sensitivity of the capacitance gradient to the interlamellar separation, ${\partial \mathord{\left/ {\vphantom {\partial {\partial d}}} \right. \kern-\nulldelimiterspace} {\partial d}}\left( {{{dC} \mathord{\left/ {\vphantom {{dC} {dz}}} \right. \kern-\nulldelimiterspace} {dz}}} \right)$, as a function of the interlamellar separation for a representative bi-lamellar liposome of height *h* = 100 nm and diameter *D* = 500 nm for three different conductivities of the internal solution. The curves plotted in Fig. 5a are obtained by just performing the derivative of the the *dC/dz* vs *d* curves in Fig. 1b. As threshold value (dashed line in Fig. 5a) we selected the sensitivity corresponding to a 5 nm variation of the interlamellar separation for an instrument with typical measuring noise of ~ 0.05 aF/nm, ${\partial \mathord{\left/ {\vphantom {\partial {\partial d}}} \right. \kern-\nulldelimiterspace} {\partial d}}\left( {{{dC} \mathord{\left/ {\vphantom {{dC} {dz}}} \right. \kern-\nulldelimiterspace} {dz}}} \right)_{ref} = 0.01\;{\text{aF/}}nm^{2}$. It is observed that in-liquid SDM is sensitive to the interlamellar separation only when relatively large internal conductivities are considered (here for σ_*lip*_ > 0.1 S/m), and only in a given specific range of values, which depends on the conductivity of the internal solution. For instance, according to the data in Fig. 5, for σ_*lip*_ = 1 S/m (close to the experimental value of σ_lip_ ~ 0.8 S/m) in-liquid SDM measurements are sensitive to interlamellar separation smaller than ~ 20 nm. Reducing the noise of the measuring instrument one could access to deeper internal lamellae (up to ~ 40 nm separation), but a limit in the depth sensitivity is always observed. This result implies that the measurements are not sensitive to the lamellae located deep inside the liposome (this fact can also be inferred from the trend of the dashed lines in Fig. 3a for increasing interlamellar separations). Therefore, strictly speaking, the lamellarity values reported here correspond to minimum lamellarity values. In practice, however, multilamellar liposomes tend to present the lamellae close packed, and hence, the estimation provided by our method is expected to constitute an excellent estimation of the actual lamellarity of the liposomes. This statement is supported by the fact that in the measurements reported here, only interlamellar separations up to ~ 10 nm have been detected, while the system sensitivity was able to detect interlamellar separations up to ~ 20 nm.

**Fig. 6:**
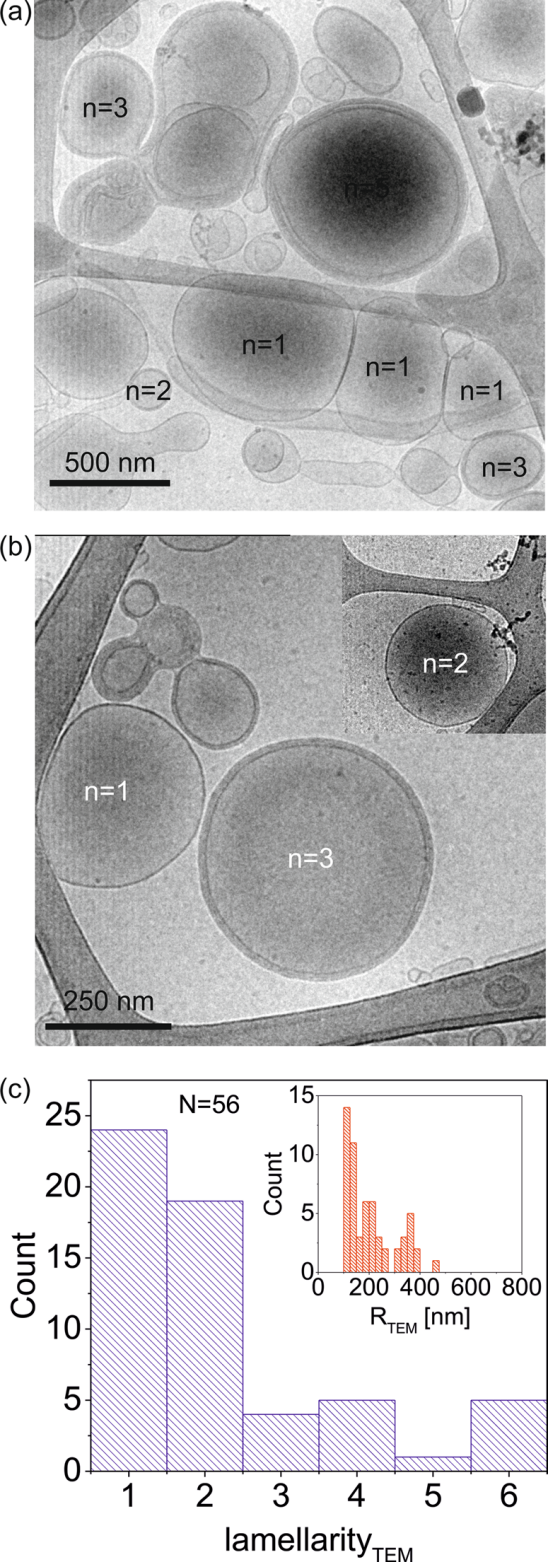
**a**, **b** Representative cryo-TEM images of non-extruded DOPC liposomes. In some of the liposomes we highlighted the lamellarity, *n*. The inset in **b** corresponds to a different image with the same scale bar than **b**. **c** Lamellarity distribution obtained after analyzing *N* = 51 liposomes with a size distribution and shape like those of the liposomes analyzed by SDM in Fig. 2 (100 nm < *R*_*TEM*_ < 800 nm, spherical shape). The inset shows the size distribution of the liposomes analyzed

The performance of SDM as single liposome lamellarity technique has been assessed against a well stablished technique like cryo-TEM. Figure 6a and b show characteristic images obtained by cryo-TEM, with the lamellarity of some liposomes highlighted. Figure 6c shows the lamellarity distribution obtained from the cryo-TEM images after analyzing *N* = 58 liposomes with radii and shape comparable to the radii and shape of the liposomes analyzed by SDM (100 nm < *R*_*TEM*_ < 800 nm, spherical shape). The distribution of sizes (inset of Fig. 6c) is also comparable (Fig. 2d). The lamellarity distribution shows a predominant presence of low lamellarity (*n* = 1, 2) liposomes (82% of the total), followed by a much less abundant (18% of the total) presence of high lamellarity liposomes (*n* ≥ 3). This result is similar to the one obtained from the SDM measurements, where 86% of the liposomes showed low lamellarity (*n* = 1, 2) and 14% a high lamellarity (*n* ≥ 3) (Fig. 3b). The cryo-TEM images seem to show a larger proportion of uni-lamellar liposomes as compared to the SDM results. We believe that this fact reflects more a different sampling of the two samples analyzed, rather than a fundamental difference.

Concerning high lamellarity liposomes (n ≥ 4) they represent roughly a 10% of the liposomes analyzed in the cryo-TEM images. Such high lamellarities were not observed in the SDM measurements. This fact reflects that SDM is sensitive to the internal structure of the liposomes up to a certain depth, which has been estimated to be ~ 20 nm in the present experiments (see Fig. 5). Therefore, for liposomes with high lamellarities, the internal lamellae sit beyond the accessible depth, and hence they become undetectable to the SDM measurements. The corresponding liposomes are then assigned to the highest lamellarity detectable, here n = 3.

**Fig. 7:**
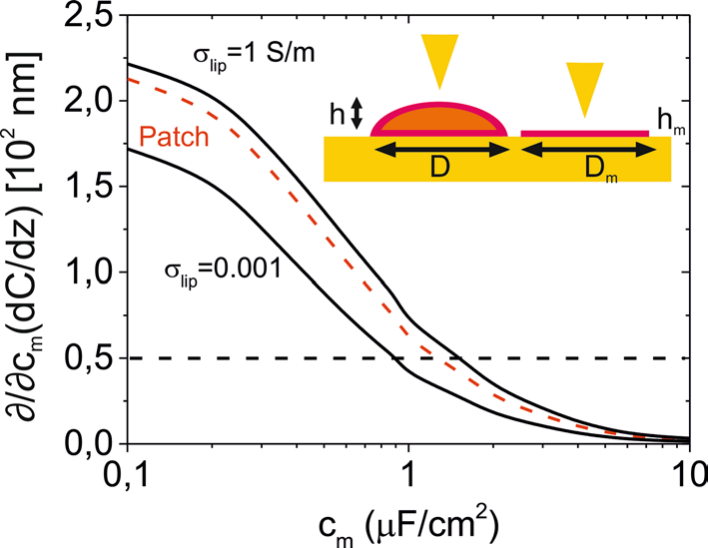
Theoretical sensitivity of the capacitance gradient to variations of the specific capacitance of the lipid bilayer membrane, *c*_*m*_, as a function of the specific capacitance for measurements performed on a uni-lamellar liposome with height *h* = 100 nm, diameter *D* = 500 nm and internal conductivities σ_*lip*_ = 0.001 S/m and 1 S/m (black continuous lines). The red dashed line corresponds to the case of a solid supported planar lipid bilayer patch of diameter *D*_*m*_ = 500 nm and thickness *h*_*m*_ = 5 nm. Parameters of the calculations: σ_*sol*_ = 0.2 S/m, *ε*_*sol*_ = *ε*_*lip*_ = 78, *R* = 30 nm, *θ* = 20°, *f*_*el*_ = 5 MHz, *z* = 50 nm. Inset: schematic representation of the two systems considered

The analysis of the liposomes' lamellarity by means of in-liquid SDM also provides the value of the lipid bilayer specific capacitance, *c*_*m*_. This value has been here identified resorting to the fact that the equivalent homogeneous dielectric constant of the liposomes, *ε*_*eq*_, shows the steepest dependence on the liposomes' height for the case of uni-lamellar liposomes. The value obtained proceeding in this way was, *c*_*m*_ ~ 0.75 µF/cm^2^, which is in remarkable agreement with the value found recently on solid supported DOPC planar lipid bilayer patches by means of the same technique (*c*_*m*_ ~ 0.70–0.8 µF/cm^2^, [41]). This result demonstrates that the lipid bilayer specific capacitance, *c*_*m*_, can be precisely measured in liposomes with the proposed approach. Remarkably, the measuring method is sensitive to the lipid bilayer specific capacitance, *c*_*m*_, in the range of values of interest for lipid bilayers *c*_*m*_ ~ 0.1–1 µF/cm^2^. To show it, in Fig. 7 we plot the sensitivity of the capacitance gradient to variations of the membrane specific capacitance, ${\partial \mathord{\left/ {\vphantom {\partial {\partial c_{m} }}} \right. \kern-\nulldelimiterspace} {\partial c_{m} }}\left( {{{dC} \mathord{\left/ {\vphantom {{dC} {dz}}} \right. \kern-\nulldelimiterspace} {dz}}} \right)$ for different internal conductivities (black continuous lines) and compare them with the threshold value determined by the instrumental noise ${\partial \mathord{\left/ {\vphantom {\partial {\partial c_{m} }}} \right. \kern-\nulldelimiterspace} {\partial c_{m} }}\left( {{{dC} \mathord{\left/ {\vphantom {{dC} {dz}}} \right. \kern-\nulldelimiterspace} {dz}}} \right)_{ref} = 0.5\;10^{2} {\text{nm}}$, corresponding to a variation of the specific capacitance of 0.1 µF/cm^2^ and to an instrumental noise of 0.05 aF/nm (see Additional file 1: S5 for additional data). This result indicates that the full range of lipid bilayer specific capacitance values of interest (*c*_*m*_ ~ 0.1–1 µF/cm^2^) can be accessed with the proposed approach. It is worth remarking that the accuracy with which *c*_*m*_ can be measured in liposomes is similar to the one that can be obtained on solid supported planar lipid bilayers, since the sensitivity ${\partial \mathord{\left/ {\vphantom {\partial {\partial c_{m} }}} \right. \kern-\nulldelimiterspace} {\partial c_{m} }}\left( {{{dC} \mathord{\left/ {\vphantom {{dC} {dz}}} \right. \kern-\nulldelimiterspace} {dz}}} \right)$ of in-liquid SDM measurements in both cases is similar (compare red dashed and solid black lines corresponding, respectively, to planar bilayers and to liposomes in Fig. 7).

This result is a consequence of the fact that the ac voltage applied on a liposome mostly drops across the lipid bilayer since it is much less polarizable than the core of the liposome due to both the high dielectric constant of the internal solution and to its conductivity. We highlight that when the specific capacitance of the lipid bilayer of the liposomes is the magnitude of interest, it is convenient to prepare the liposomes by using a method that guarantees the presence of enough uni-lamellar liposomes (e.g. by using extrusion or inverted emulsion methods).

The theoretical analysis of the in-liquid SDM measurements performed in the present work has been carried out by using finite element numerical calculations. While this is necessary in order to obtain accurate quantitative values of the tip-liposome capacitance gradient, *dC/dz*, it can also make complex the understanding of the underlying physics. To facilitate the analysis, we propose a phenomenological analytical expression for *dC/dz*, following our previous analysis of the tip-nanoparticle electrical interaction in air environment [25]. The phenomenological tip-liposome capacitance gradient relation is of the form

1 $$\frac{dC}{{dz}} \approx \alpha + \beta \log \left( {\left| {\varepsilon_{eq}^{*} } \right|} \right)$$

**Fig. 8:**
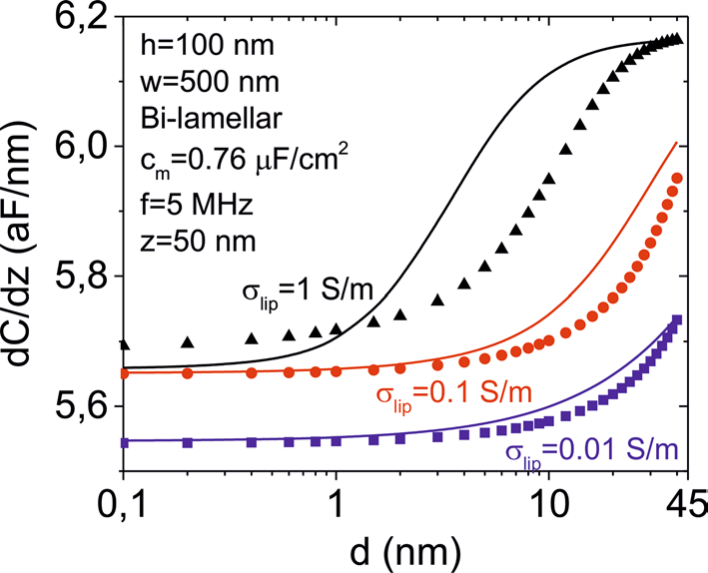
(Continuous lines) Tip-bi-lamellar liposome capacitance gradient as a function of the interlamellar separation obtained from Eq. (1) with *ε*_eq_^*^ given by the expression corresponding to a spheroidal bi-lamellar liposome in an external uniform ac electric field of height *h* = 100 nm, diameter *D* = 500 nm, bilayer thickness *t*_*m*_ = 3.5 nm, and bilayer dielectric constant *ε*_*m*_ = 3 (corresponding to a specific membrane capacitance *c*_*m*_ = 0.76 µF/cm^2^), for different conductivities of the internal solution, σ_*lip*_. The internal and external solutions are assumed to have the same dielectric constant, *ε*_*ip*_ = *ε*_sol_ = 78, while the external conductivity is σ_*sol*_ = 0.2 µS/m. The phenomenological parameters *α* and *β* in Eq. (1) have been set to *α* = 3.53 aF/nm and *β* = 1.7 aF/nm in order to best reproduce qualitatively the capacitance gradient values numerically calculated for an adsorbed bi-lamellar liposome with a spherical cap geometry of the same dimensions (symbols, same data as in Fig. 1c)

Here, *ε*_eq_^*^ is the equivalent homogeneous complex permittivity of a spheroidal liposome in a uniform external ac electric field [47, 48] with the height and width being equal to the ones of the absorbed liposome (see Additional file 1: S3 for explicit expressions of ε_eq_^*^ for uni- and bi-lamellar liposomes). Here, *α* and *β* are two phenomenological parameters that depend on the size of the liposome and of the tip, and on the tip-sample distance, among other parameters. Figure 8 (continuous lines) shows the prediction of Eq. (1) for the tip-bi-lamellar liposome system analyzed numerically in Fig. 1c (for an easier reference the numerically calculated data reported in Fig. 1c, are plotted again in Fig. 8 as symbols). The best matching between the numerically calculated data and the ones given by the anlytical expression in Eq. (1) has been obtained by taking *α* = 3.53 aF/nm and *β* = 1.7 aF/nm. It is seen that the analytical expression predicts reasonably well the dependence of the capacitance gradient on the interlamellar separation for the different internal conductivities. It can be shown that Eq. (1) also correctly predicts qualitatively the dependence of *dC/dz* on some other system parameters like the bilayer specific capacitance, *c*_*m*_, or the frequency of the external applied voltage, *f*_*el*_ (see Additional file 1: S6).

## Discussion

We have measured the dielectric properties of sub-micrometric single liposomes adsorbed on a planar electrode by means of in-liquid scanning dielectric microscopy in force detection mode. For each liposome we have determined the equivalent homogeneous dielectric constant, *ε*_*eq*_, and demonstrated that this parameter can be used to determine the lamellarity of the liposomes, the separation between the lamellae in the case of bi-lamellar liposomes, *d*, and the specific capacitance of the lipid bilayer, *c*_*m*_. For non-extruded DOPC liposomes we have detected liposomes containing up to 3 lamellae, with the bi-lamellar liposomes being the more abundant ones (Fig. 3b). Concerning the separation between lamellae, in the case of bi-lamellar liposomes we have found it to be small (*d* < 10 nm), indicating that the lamellae are close packed. As the lamellarity increases (*n* > 2), the number of possible combinations of interlamellar separations increases, and the interlamellar separation cannot be unambiguously determined. Besides this fact, the sensitivity to lamellarity, and even more, to interlamellar separation, decreases.

With the proposed approach the lamellarity of single liposomes has been determined in a non-invasive and label-free way, what constitutes an advantage with respect to other existing techniques used to determine the lamellarity of single liposomes, such as electron and fluorescence microscopies or mechanical probing [18, 19]. With respect to cryo-transmission electron microscopy or freeze-fracture electron microscopy, it offers the advantage of being non-destructive, in the sense that it enables determining the lamellarity and performing further functional analyses on a given liposome (e.g. to determine other biophysical properties or investigate biochemical reactions and time dependent processes). The main disadvantage is that it is not sensitive to liposomes of high lamellarity (n ≥ 4) or to very small liposomes (*R*_*eq*_ < 100 nm). This limitation is not too relevant to determine the distribution of lamellarities since high lamellarity liposomes are not very abundant among sub-micrometric sized liposomes of interest for SDM, and because very small-scale liposomes are usually uni-lamellar. With respect to fluorescence microscopy, it offers the advantage of being label-free and to provide information on the lipid bilayer specific capacitance. The specific capacitance of a lipid bilayer provides information complementary to the polarity that can be obtained, for instance, by using environmental sensitive probes [49–51]. Finally, with respect to mechanical techniques such as micropipette aspiration [20, 21] and force spectroscopy AFM [11], the present approach offers the advantage of being non-contact, and hence less invasive, and applicable to both gel and liquid phase liposomes. For liquid phase liposomes the detection of deformations or multiple rupture events by mechanical methods can be problematic due to the very soft nature of the lipid bilayers in these cases (stiffness ~ 1 mN/m) [46]. Besides this, the present approach offers dielectric information on the liposome (e.g. specific lipid bilayer capacitance) complementary to the mechanical information obtained by means of the mechanical techniques.

Finally, we note that in the present work measurements have been done in a low conductivity external solution (nominally σ_*sol*_ ~ 0.2 µS/m, although in practice probably higher due to salt contamination). In-liquid SDM measurements can be carried out also in solutions of higher conductivity, as reported earlier [26]. In this case higher measuring frequencies become necessary. Typically, for solutions up to 10 mM ionic concentration measurements can be performed for frequencies lower than 100 MHz, following the methods described in the present work. For higher concentrations, frequencies in the GHz range would be needed; this would require some specific adaptations to the measuring set-ups.

The proposed methodology is expected to be applicable to the study of the dielectric and structural properties of other lipidic sub-micrometric systems, like proteoliposomes or lipoplexes or to lipidic systems showing non-lamellar structures (e.g. cubic or hexagonal). However, in each case a specific study on the sample preparation protocol for in-liquid SDM imaging should be undertaken, as well as, the development of the corresponding theoretical analysis in order to determine what specific properties the technique is sensitive to.

## Conclusions

We have shown that the lamellarity of single liposomes can be measured in a non-invasive and label-free way by means of in-liquid scanning dielectric microscopy. The measurements provide, in addition, information on the interlamellar separation in bi-lamellar liposomes and on the specific capacitance of the lipid bilayer membrane. For non-extruded DOPC liposomes we have found that for equivalent radii larger than ~ 400 nm liposomes are at least tri-lamellar, while for radii between ~ 250 and 400 nm they are bi-lamellar. For radii below ~ 250 nm both uni- and bi-lamellar liposomes are present. The interlamellar separations of the bi-lamellar liposomes have been found to be below ~ 10 nm. Concerning the lipid bilayer specific capacitance, we obtained a value *c*_*m*_ ~ 0.75 µF/cm^2^, in good agreement with the value obtained on solid supported planar lipid bilayers. We have shown that *c*_*m*_ in liposomes can be measured with a similar accuracy than on planar lipid bilayers. The proposed approach can be applied to both liquid and gel phase liposomes, since it is essentially a non-contact technique. Present results open interesting routes for the label-free analysis and characterization of single liposomes and of nanovesicles in general.

## Materials and methods

### Liposome preparation and adsorption on metallic substrates

DOPC (1,2-dioleoyl-sn-glycero-3-phosphocholine) multi-lamellar liposomes were obtained by the hydration method, without a further step of extrusion, in a salt-reach buffer composed of TRIS 20 mM, KCl 100 mM. For the preparation of the liposomes, chloroform and methanol, HPLC grade, were purchased from Sigma Aldrich; high purity water (18.2 MΩ·cm) was obtained with a MilliQ water purification system (Millipore, Billerica, MA); DOPC specified as R99% pure, was obtained in powder form (Avanti Polar Lipids (Merk)) and used without further purification. The liposomes were prepared as follows: DOPC was first dissolved in a chloroform/methanol (3:1) (v/v) solution to a final lipid concentration of 10 mM. Then, the solvent was evaporated under a nitrogen stream with constant rotation of the vial. The vial was kept in vacuum for 6–8 h to ensure the absence of organic solvent traces. The dry lipid film was then resuspended in TRIS 20 mM, KCl 100 mM to a final concentration of 0.1 mM. The liposomes were spontaneously formed under these conditions and stored at 2–8 °C, always protected from light, and used within 1–2 days. A drop of 60 μL of DOPC liposomes suspension was added to a flat functionalized gold substrate at room temperature (25 °C) and incubated for 5 min. The salt-rich buffer and the short incubation time at 20 °C withhold the rupture of most liposomes onto the surface. The multi-lamellar nature also contributes to that. Afterwards, the substrate was rinsed several times with milliQ water to remove the excess of vesicles in suspension and to adapt the media to the imaging requirements of SDM (low salt concentration). The flat gold substrates were produced by the mica replica method (MicroFab Space, IBEC, Spain) and functionalized with self-assembled monolayers (SAMs) made of 2-Mercaptoethanol 99.0% (Sigma-Aldrich). The SAMs were prepared by incubating the gold substrates in a 1 mM solution of the thiols overnight at 2–8 °C, protecting the vial from light and from oxidation by filling it with nitrogen before storage. Gold was selected for its excellent conductive properties, while the alcoholic moiety terminating the thiol molecules was chosen to make the surface more hydrophilic, promoting the interaction with the lipid polar heads and, hence, the adsorption of the liposomes.

### In-liquid SDM measurements

In-liquid SDM measurements were done by following the methodology originally proposed in Ref. [25] for measurements on nanoparticles and viruses in air, adapted to the liquid environment in Refs. [26, 42]. In a nutshell, an amplitude modulated ac voltage of frequency *f*_*el*_ and modulation frequency *f*_*mod*_ is applied between the conductive probe of an AFM system and the substrate in an electrolyte solution. The cantilever oscillation amplitude at the *f*_*mod*_ frequency is then recorded, from where the tip-liposome capacitance gradient, *dC/dz*, can be extracted [42]. Standard SDM images in the two-pass constant-height mode at different heights as well as *dC/dz* approach curves on bare parts of the substrate have been acquired for tip calibration purposes, as detailed in Ref. [25]. The specific experimental implementation followed here is the same as in Ref. [41], to which we refer for further experimental details.

### Finite element numerical calculations

We quantitatively analysed the SDM images following the methodology developed in Ref. [25], adapted to the liquid environment as in Refs. [40, 42]. We calculated theoretical *dC/dz* values for the corresponding tip-liposome model by solving the currents model implemented in the AC/DC Electrostatic module of COMSOL Multiphysics (Comsol Inc.). We did not include ionic diffusive effects, since they can be neglected at the frequencies of the in-liquid SDM measurements [42, 52]. The probe was modelled as a cone with a tangent sphere as in previous works [25, 53, 54], and it included the presence of an interfacial capacitance [42]. The geometry of the liposomes has been modelled as a cap of height *h* and width *D* and shape described by the revolution of the function *Z(X)* = *h-a (X-X*_*0*_*)*^*b*^, where *X*_*0*_ is the centre of the liposome, *b* = *ln(2)/ln(D/FWHM)* and *a* = *h/(D/2)*^*b*^, with *FWHM* being the full width at half maximum. By including the adjustment of the profile at the FWHM point, the geometry generated by this function adapts better to the actual measured geometry than a simpler spherical cap (see Additional file 1: S7). The parts of the substrate not covered by the liposomes have an interfacial capacitance, *c*_*subs*_, as detailed in previous works [40, 42]. To determine the equivalent homogeneous dielectric constant of the liposomes, *ε*_*eq*_, we considered, as in previous works with virus particles [25] and single bacterial cells [28, 29], a model in which the whole liposome is assumed to have a uniform dielectric constant, *ε*_*eq*_, with the shape and size obtained from the topographic image (bottom insert of Fig. 3a). To describe the properties of uni-lamellar liposomes we considered a core–shell model (top insert of Fig. 3a), similar to the one used in those previous works [25, 28, 29] but including the effects of the conductivity of the core. In the models, a membrane of thickness, *d*_*m*_, and dielectric constant, *ε*_*m*_, surrounds a core with dielectric constant *ε*_*lip*_ and conductivity σ_*lip*_. For small membrane thicknesses, the dielectric response of the liposome only depends on the membrane specific capacitance, *c*_*m*_ = *ε*_*0*_*ε*_*m*_*/d*_*m*_, which is the parameter that we reported. To describe bi-lamellar liposomes, we added to the uni-lamellar model a concentric internal lamella at a distance *d* from the external membrane (see Fig. 1b). The electric force acting on the probe was determined by integration of the Maxwell stress tensor over the conical part of the tip (integration on the cantilever was avoided to reduce the numerical noise). Direct cantilever effects were modelled phenomenologically through a constant offset, *C'*_*off*_ [42]. A sinusoidal voltage was used in the simulations, so that the capacitance gradient values were obtained from the amplitude of the 2ω calculated force harmonic, *F*_*2*ω*,num*_ = *1/4 dC/dz v*_*0*_^*2*^.

### Extraction of the equivalent homogeneous dielectric constant of the liposomes

To extract the equivalent homogeneous dielectric constant of the liposomes, *ε*_*eq*_*,* we considered the homogeneous dielectric model described above and followed the approach detailed in Ref. [25]. We calibrated the tip geometry and substrate interfacial capacitance by using *dC/dz* curves acquired on the metallic substrate [25, 53, 54]. The fittings were done by keeping the half cone angle, cone height and cantilever thickness fixed to manufacturer values (*θ =* 21°, *H* = 12.5 µm, *W* = 3 µm,) and by fixing the tip interfacial capacitance to the value *c*_*tip*_ ~ 2.7 µF/cm^2^ derived in Ref. [42]. The cantilever length was kept to *L* = 3 µm, which is a reasonable value to include indirect effects with a cantilever disc model [54]. The voltage reduction factor, *α*, [42] was found to be here *α* = 1.24. The result of the fitting process provided the tip radius, *R*, the substrate interfacial capacitance, *c*_*sub*_ and the capacitance gradient offset, *C'*_*off*_. With the obtained parameters, we calculated capacitance gradient values, *dC/dz*, for the tip located on top of the liposomes at their centre at different distances *z* from the substrate and fitted them to the experimental values extracted from the SDM images acquired at different heights with the equivalent dielectric constant of the liposome, *ε*_*eq*_, as the single fitting parameter, as detailed in Ref. [25]. The analysis was done with a custom-made software written in Matlab (Mathworks inc.) linked to the COMSOL Multiphysics software.

### Cryo-TEM liposome imaging

3.9 µl of liposome suspension were blotted onto 400 mesh holey carbon grids (© Micro to Nano 2021) previously glow discharged in a PELCO easiGlow glow discharge unit. Next, the sample was plunged into liquid ethane (− 180 °C) by means of a Leica EM GP cryo workstantion and subsequently transferred and visualized in a Jeol JEM-2011 TEM electron microscope operating at 200 kV. Samples were maintained at − 180 °C during their observation and captures were obtained with a Gatan CCD 895 ultrascan camera. The dimensions of single liposomes were obtained from the TEM images by using the Measure tool of ImageJ, which provides the area, A_TEM_, perimeter, L_TEM_ and roundness, r, among other factors, where *r* = *4·A*_*TEM*_*/π·a*_*TEM*_^*2*^, with *a*_*TEM*_ being the major axis. The equivalent spherical radii of the liposomes, *R*_*TEM*_, has been determined from the perimeter, *L*_*TEM*_ = *2πR*_*TEM*_. Lamellarity was settled by digital zooming the images and measuring the thickness of the membranes. For statistical analysis only liposomes with radius, *R*_*TEM*_, in the range 100–800 nm and roundness, *r* > 0.8 were considered.

## Supplementary Information

**Additional file 1.** Comparison of the distribution of the spherical equivalent radii of the liposomes assuming a constant area and volume. Additional data for Fig. 2. Equivalent complex permittivity of uni- and bi-lamellar core–shell spheroidal liposomes in an external uniform ac electric field. S4. Calibration curve on the substrate and extracted tip geometry. S5. Dependence of the capacitance gradient on the specific membrane capacitance. S6. Additional data for Fig. 7. S7. Geometrical model for the adsorbed liposomes.

## Data Availability

The datasets used and/or analysed during the current study are available from the corresponding author on reasonable request.

## Abbreviations

SDM: Scanning dielectric microscopy
DNA: Deoxyribonucleic acid
DOPC: 1,2-Dioleoyl-sn-glycero-3-phosphocholine
AFM: Atomic force microscopy
SAM: Self-assembled monolayer
FWHM: Full width at half maximum
TEM: Transmission electron microscopy
