# Supplementary material for: Dielectric properties and lamellarity of single liposomes measured by in-liquid scanning dielectric microscopy
Source: J Nanobiotechnology. 2021 Jun 3;19:167. doi: 10.1186/s12951-021-00912-6 (PMC8176598; doi:10.1186/s12951-021-00912-6)
Supplement: Supplementary file 1 — Additional file 1. Comparison of the distribution of the spherical equivalent radii of the liposomes assuming a constant area and volume. Additional data for Fig. 2. Equivalent complex permittivity of uni- and bi-lamellar core–shell spheroidal liposomes in an external uniform ac electric field. S4. Calibration curve on the substrate and extracted tip geometry. S5. Dependence of the capacitance gradient on the specific membrane capacitance. S6. Additional data for Fig. 7. S7. Geometrical model for the adsorbed liposomes. [file 12951_2021_912_MOESM1_ESM.pdf]

Supporting Information

for

Dielectric Properties and Lamellarity of Single Liposomes Measured by In-Liquid Scanning Dielectric Microscopy

Martina Di Muzio[1], Ruben Millan-Solsona[1,2], Aurora Dols-Perez[1], Jordi H. Borrell[3,4], Laura Fumagalli[5,6], Gabriel Gomila[*1,2]

[1]*Institut de Bioenginyeria de Catalunya (IBEC), The Barcelona Institute of Science and Technology, c/ Baldiri i Reixac 11-15, 08028, Barcelona, Spain.*

[2]*Departament d'Enginyeria Electrònica i Biomèdica, Universitat de Barcelona, C/ Martí i Franquès 1, 08028, Barcelona, Spain*

[3]*Secció de Fisicoquímica, Facultat de Farmàcia i Ciències de l'Alimentació, Universitat de Barcelona, 08028, Barcelona, Spain*

[4]*Institute of Nanoscience Nanotechnology, Universitat de Barcelona, 08028, Barcelona, Spain*

[5]*Department of Physics and Astronomy, University of Manchester, Manchester M13 9PL, UK.*

[6]*National Graphene Institute, University of Manchester, Manchester M13 9PL, UK.*

*Corresponding author: ggomila@ibecbarcelona.eu

S1. Comparison of the distribution of the spherical equivalent radii of the liposomes assuming a constant area and volume

The surface area, S_{cap}, and volume, V_{cap}, of a spherical cap of diameter D and height h are given, respectively, by (1)

$$S_{cap} = \pi\left[\left(\frac{D}{2}\right)^2 + h^2\right] + \pi\left(\frac{D}{2}\right)^2; \qquad V_{cap} = \frac{1}{6}\pi h\left[3\left(\frac{D}{2}\right)^2 + h^2\right]. \tag{S1}$$

The surface area, S_{sphere}, and volume, V_{sphere}, of a sphere of radius R are given, respectively, by (1)

$$S_{sphere} = 4\pi R^2; \qquad V_{sphere} = \frac{4}{3}\pi R^3. \tag{S2}$$

Accordingly, by equating the surfaces and volumes in Eqs. (S1) and (S2), we obtain the equivalent spherical radius assuming constant the surface area or the volume of the liposome, $R_{eq,S}$ and $R_{eq,V}$, respectively,

$$R_{eq,S} = \frac{1}{2}\sqrt{\left(2\left(\frac{D}{2}\right)^2 + h^2\right)}; \qquad R_{eq,V} = \frac{1}{2}\sqrt[3]{h\left[3\left(\frac{D}{2}\right)^2 + h^2\right]}. \tag{S3}$$

$R_{eq,S}$ in Eq. (S3) has been used to generate the distribution of equivalent radii in Fig. 2d (reproduced in Fig. S1, red bars, for easier reference). The distribution of radii obtained by using $R_{eq,V}$ in Eq. (S3) is shown in Fig. S1 (blue bars).

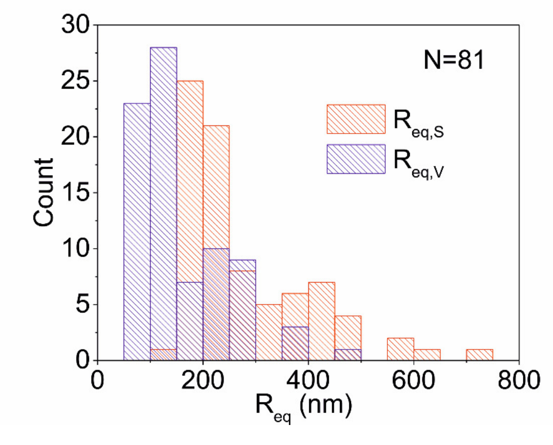

Figure S1: Distribution of the equivalent spherical radii for the liposomes in Fig. 2a, assuming a constant surface or volume area during the adsorption (red and blue bars, respectively).

S2. Additional data for Fig. 2.

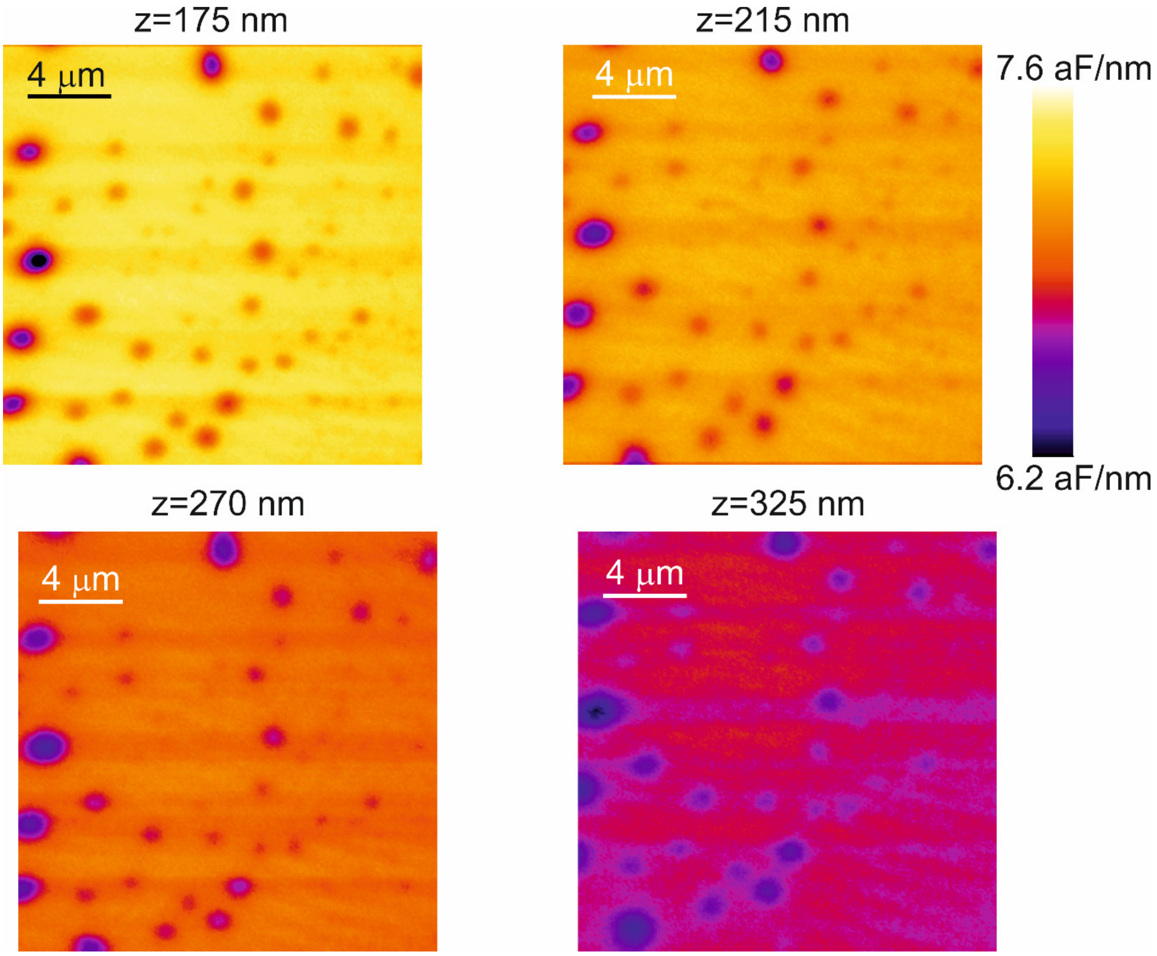

Figure S2. In-liquid SDM images of the liposome sample in Fig. 2 acquired at four different tip-substrate distances. The image at z=270 nm corresponds to the image in Fig. 2e of the main manuscript. Note that the color palette of the scale is the same as in Fig. 2, but the range of values covered is different, so that we can visualize all the images in a common color scale.

S3. Equivalent complex permittivity of uni- and bi-lamellar core-shell spheroidal liposomes in an external uniform ac electric field.

For a uni-lamellar core-shell spheroidal liposome of height h, width D and shell thickness t_m, in a uniform external ac electric field, the equivalent homogeneous complex permittivity, ε_{eq}^{*}, in the direction of the external electric field is given by (2), (3)

$$\varepsilon_{eq}^{*} = \varepsilon_m \frac{\left(\dfrac{\left(\frac{D}{2}\right)^2 \frac{h}{2}}{\left(\frac{D-2t_m}{2}\right)^2 \frac{h-2t_m}{2}}\right) + \dfrac{\left(\varepsilon_{lip}^{*}-\varepsilon_m\right)\left(1-L_z\left(\frac{D}{2},\frac{h}{2}\right)\right)}{\left(\varepsilon_{lip}^{*}-\varepsilon_m\right)L_z\left(\frac{D-2t_m}{2},\frac{h-2t_m}{2}\right)+\varepsilon_m}}{\left(\dfrac{\left(\frac{D}{2}\right)^2 \frac{h}{2}}{\left(\frac{D-2t_m}{2}\right)^2 \frac{h-2t_m}{2}}\right) - \dfrac{\left(\varepsilon_{lip}^{*}-\varepsilon_m\right)L_z\left(\frac{D}{2},\frac{h}{2}\right)}{\left(\varepsilon_{lip}^{*}-\varepsilon_m\right)L_z\left(\frac{D-2t_m}{2},\frac{h-2t_m}{2}\right)+\varepsilon_m}} \tag{S4}$$

Here

$$\varepsilon_{lip}^{*} = \varepsilon_{lip} + i\frac{\sigma_{lip}}{\varepsilon_0 \omega} \tag{S5}$$

is the complex permittivity of the solution entrapped inside the liposome, with ε_{lip} and σ_{lip} being its permittivity and conductivity (here we do not consider polarization losses). ω is the angular frequency of the ac electric field and ε_m the lipid bilayer permittivity, assumed to be real (without losses or conductivity). Finally, L_z is the polarization factor given by (3)

$$L_z\left(a_x,a_z\right) = \frac{1}{2\left[\sqrt{1-\left(\frac{a_z}{a_x}\right)^2}\right]^3}\left[2\sqrt{1-\left(\frac{a_z}{a_x}\right)^2} - \pi\frac{a_z}{a_x} + 2\frac{a_z}{a_x}\arctan\left(\frac{\frac{a_z}{a_x}}{\sqrt{1-\left(\frac{a_z}{a_x}\right)^2}}\right)\right]; \quad a_z < a_x \tag{S6}$$

In the spherical limit, one has D=h and L_z=1/3, and one recovers the well-known relation for the equivalent homogeneous permittivity of a spherical core-shell particle (2). In the homogeneous limit, when $\varepsilon_{lip}^{*}= \varepsilon_m$, one has $\varepsilon_{eq}^{*}= \varepsilon_m$, as it should be. Note that the equivalent homogeneous complex permittivity of a core-shell uni-lamellar liposome depends on the size of the liposome.

For the case of a bi-lamellar liposome, one applies recursively Eq. (S6) following the scheme shown in Fig. S1 as described elsewhere (2).

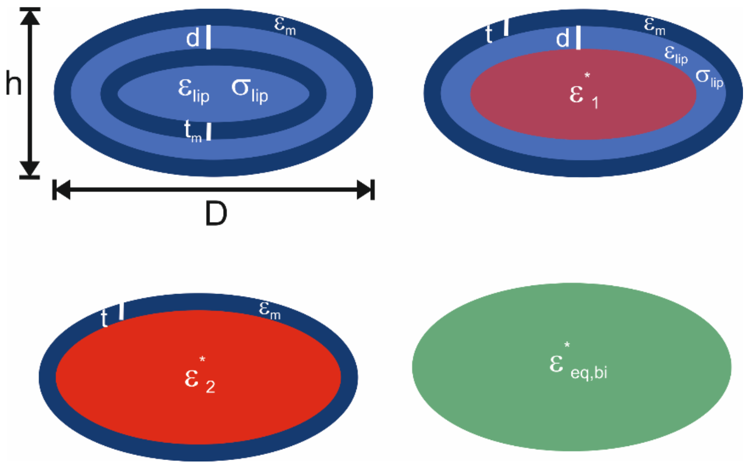

Figures S3. Recursive approach to determine the equivalent complex permittivity of a bi-lamellar spheroidal liposome.

One obtains,

$$\varepsilon^*_{eq,bi} = \varepsilon_m \frac{\dfrac{\left(\frac{D}{2}\right)^2 \frac{h}{2}}{\left(\frac{D-2t_m}{2}\right)^2 \frac{h-2t_m}{2}} + \dfrac{\left(\varepsilon^*_2 - \varepsilon_m\right)\left(1 - L_z\left(\frac{D}{2}, \frac{h}{2}\right)\right)}{\left(\varepsilon^*_2 - \varepsilon_m\right) L_z\left(\frac{D-2t_m}{2}, \frac{h-2t_m}{2}\right) + \varepsilon_m}}{\dfrac{\left(\frac{D}{2}\right)^2 \frac{h}{2}}{\left(\frac{D-2t_m}{2}\right)^2 \frac{h-2t_m}{2}} - \dfrac{\left(\varepsilon^*_2 - \varepsilon_m\right) L_z\left(\frac{D}{2}, \frac{h}{2}\right)}{\left(\varepsilon^*_2 - \varepsilon_m\right) L_z\left(\frac{D-2t_m}{2}, \frac{h-2t_m}{2}\right) + \varepsilon_m}} \qquad \text{(S7)}$$

where

$$\varepsilon^*_2 = \varepsilon^*_{lip} \frac{\dfrac{\left(\frac{D-2t_m}{2}\right)^2 \frac{h-2t_m}{2}}{\left(\frac{D-2t_m-2d}{2}\right)^2 \frac{h-2t_m-2d}{2}} + \dfrac{\left(\varepsilon^*_1 - \varepsilon^*_{lip}\right)\left(1 - L_z\left(\frac{D-2t_m}{2}, \frac{h-2t_m}{2}\right)\right)}{\left(\varepsilon^*_1 - \varepsilon^*_{lip}\right) L_z\left(\frac{D-2t_m-2d}{2}, \frac{h-2t_m-2d}{2}\right) + \varepsilon^*_{lip}}}{\dfrac{\left(\frac{D-2t_m}{2}\right)^2 \frac{h-2t_m}{2}}{\left(\frac{D-2t_m-2d}{2}\right)^2 \frac{h-2t_m-2d}{2}} - \dfrac{\left(\varepsilon^*_1 - \varepsilon^*_{lip}\right) L_z\left(\frac{D-2t_m}{2}, \frac{h-2t_m}{2}\right)}{\left(\varepsilon^*_1 - \varepsilon^*_{lip}\right) L_z\left(\frac{D-2t_m-2d}{2}, \frac{h-2t_m-2d}{2}\right) + \varepsilon^*_{lip}}} \qquad \text{(S8)}$$

and

$$\varepsilon_1^* = \varepsilon_m \frac{\dfrac{\left(\dfrac{D-2t_m-2d}{2}\right)^2 \dfrac{h-2t_m-2d}{2}}{\left(\dfrac{D-4t_m-2d}{2}\right)^2 \dfrac{h-4t_m-2d}{2}} + \dfrac{\left(\varepsilon_{lip}^* - \varepsilon_m\right)\left(1 - L_z\left(\dfrac{D-2t_m-2d}{2}, \dfrac{h-2t_m-2d}{2}\right)\right)}{\left(\varepsilon_{lip}^* - \varepsilon_m\right) L_z\left(\dfrac{D-4t_m-2d}{2}, \dfrac{h-4t_m-2d}{2}\right) + \varepsilon_m}}{\dfrac{\left(\dfrac{D-2t_m-2d}{2}\right)^2 \dfrac{h-2t_m-2d}{2}}{\left(\dfrac{D-4t_m-2d}{2}\right)^2 \dfrac{h-4t_m-2d}{2}} - \dfrac{\left(\varepsilon_{lip}^* - \varepsilon_m\right) L_z\left(\dfrac{D-2t_m-2d}{2}, \dfrac{h-2t_m-2d}{2}\right)}{\left(\varepsilon_{lip}^* - \varepsilon_m\right) L_z\left(\dfrac{D-4t_m-2d}{2}, \dfrac{h-4t_m-2d}{2}\right) + \varepsilon_m}} \quad \text{(S9)}$$

Equation (S9) depends on the size of the liposomes (*h* and *D*), as well as on the interlamellar separation, *d*.

S4. Calibration curve on the substrate and extracted tip geometry.

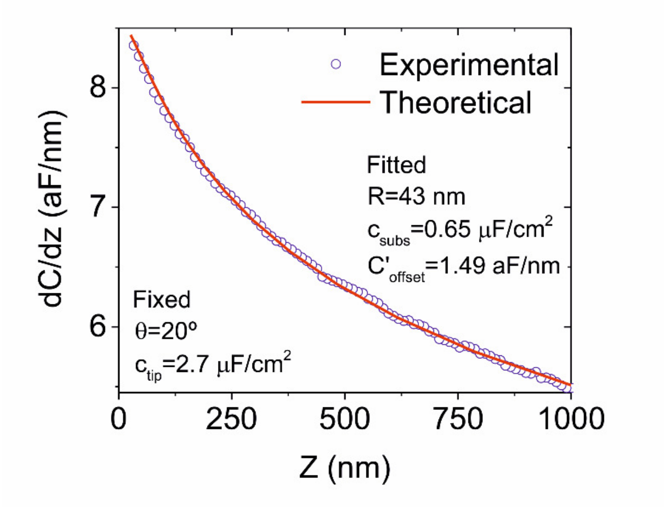

Figure S4. (Symbols) Capacitance gradient approach curve acquired on a bare part of the functionalized metallic substrate. (Continuous line) Fitted theoretical curve obtained from a substrate-tip model. The parameters fitted are the tip radius, R=43 nm, the substrate interfacial capacitance, c_{subs}=0.65 μF/cm^2 and the capacitance gradient offset C'_{offset}=1.49 aF/nm. The tip parameters that have been kept fixed are: the half cone angle, θ=20º, tip interfacial capacitance, c_{tip}=2.7 μF/cm^2, cone height, H=12.5 μm, cantilever thickness, W=3 μm and cantilever length, L_c=3 μm. A global renormalization factor α=1.24 has been applied to account for potential losses and uncertainties in the photodiode sensitivity. The renormalization factor does not affect the specific capacitance values extracted.

S5. Dependence of the capacitance gradient on the specific membrane capacitance.

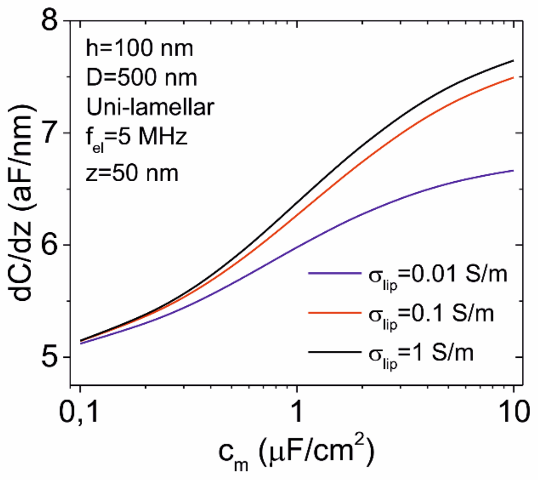

Figure S5. Numerically calculated dependence of the tip-liposome capacitance gradient, *dC/dz*, with the specific capacitance of the lipid bilayer, c_m, for a tip-uni-lamellar liposome model, for three different conductivity values of the internal media, σ_{lip}=0.01 S/m, 0.1 S/m, 1 S/m. The dependence is roughly logarithmic. Parameters: same as in Fig. 1c.

S6. Additional data for Fig. 7.

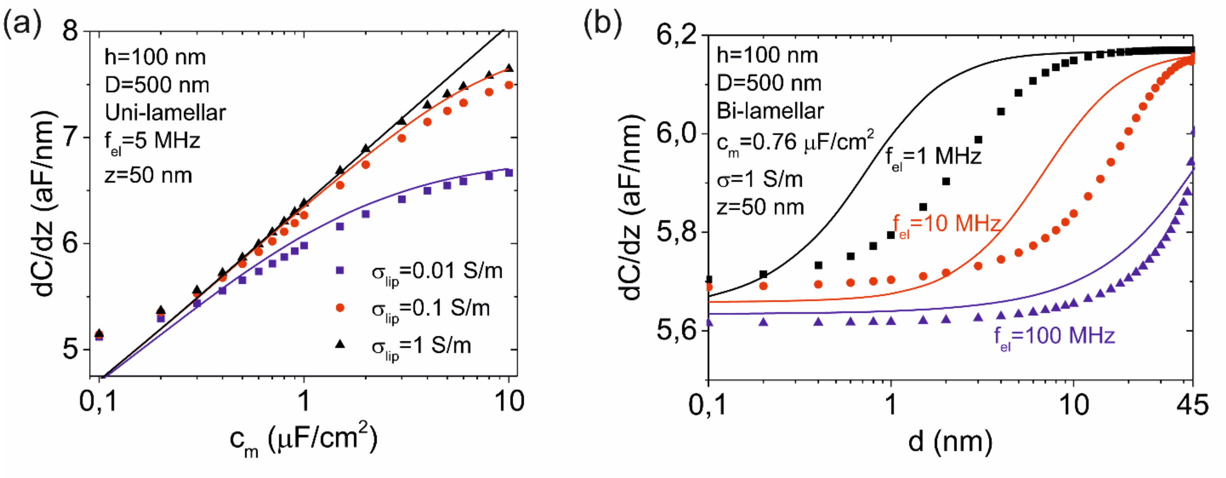

Figure S6. (a) (continuous lines) Capacitance gradient as a function of the specific capacitance of the lipid bilayer predicted by Eq. (1) of the main text, with the equivalent complex permittivity calculated with Eq. (S4) for a uni-lamellar liposome of height h=100 nm, width D=500 nm, bilayer thickness t_m=3.5 nm, bilayer dielectric constant, ε_m=3, and an aqueous internal solution with dielectric constant ε_{lip}=78, with three different conductivities σ_{lip}=0.01 S/m, 0.1 S/m, 1 S/m. The frequency of the external ac voltage is 5 MHz. The phenomenological parameters appearing in Eq. (1) have been set to α=3.53 aF/nm and β=1.7 aF/nm, to best fit the numerical calculated values represented by the symbols and corresponding to a tip-adsorbed liposome system of the same width and height. Note that the geometry of the adsorbed liposome corresponds approximately to a spherical cap, while the geometry used to derive Eq. (S4) corresponds to a full spheroid. (b) (continuous lines) Idem for the capacitance gradient of a bi-lamellar liposome as a function of the inter-lamellar separation and fixed lipid bilayer specific capacitance, c_m=0.76 μF/cm^2 and conductivity σ_{lip}=1 S/m (continuous lines) for three different frequencies f_{el}=1 MHz, 10 MHz and 100 MHz. The symbols represent the finite element numerically calculated values for the corresponding tip-adsorbed bi-lamellar liposome.

S7. Geometrical model for the adsorbed liposomes.

We have modelled the cap geometry of the adsorbed liposomes by the axial revolution of a function of the form

$$Z(X) = h - a|X - X_0|^b \qquad \text{(S10)}$$

where X_0 is the center of the liposome (position of the maximum) and

$$b = \frac{\log(2)}{\log\left(\frac{D}{FWHM}\right)}; \quad a = \frac{h}{\left(\frac{D}{2}\right)^b} \qquad \text{(S11)}$$

By substituting Eqs. (S11) into Eq. (S10), one has that the shape is described by the revolution of the function

$$Z(X) = h\left(1 - \left(\frac{2|X - X_0|}{D}\right)^{\frac{\log(2)}{\log\left(\frac{D}{FWHM}\right)}}\right) \qquad \text{(S12)}$$

This geometry is determined from three parameters of the topographic image, the height, h, the width, D, and the full width at half maximum $FWHM$. A spherical cap geometry also provides a good description of the measured topography, although, since it is determined from only two parameters of the topography (the height, h, and the width, D) it is slightly less accurate. Explicitly, the spherical cap geometry is given by

$$Z_{sph}(X) = h - R_c + \sqrt{R_c^2 - (X - X_0)^2} \qquad \text{(S13)}$$

where R_c is the radius of curvature, which for a spherical cap is given by (1)

$$R_c = \frac{(D/2)^2 + h^2}{2h} \qquad \text{(S14)}$$

In Fig. S7 we compare the predictions of the two geometrical models with the measured topography of four adsorbed liposomes spanning the full range of sizes considered. In general, both models describe correctly the measured topography, but the phenomenological model adjusts better the profiles for larger liposomes.

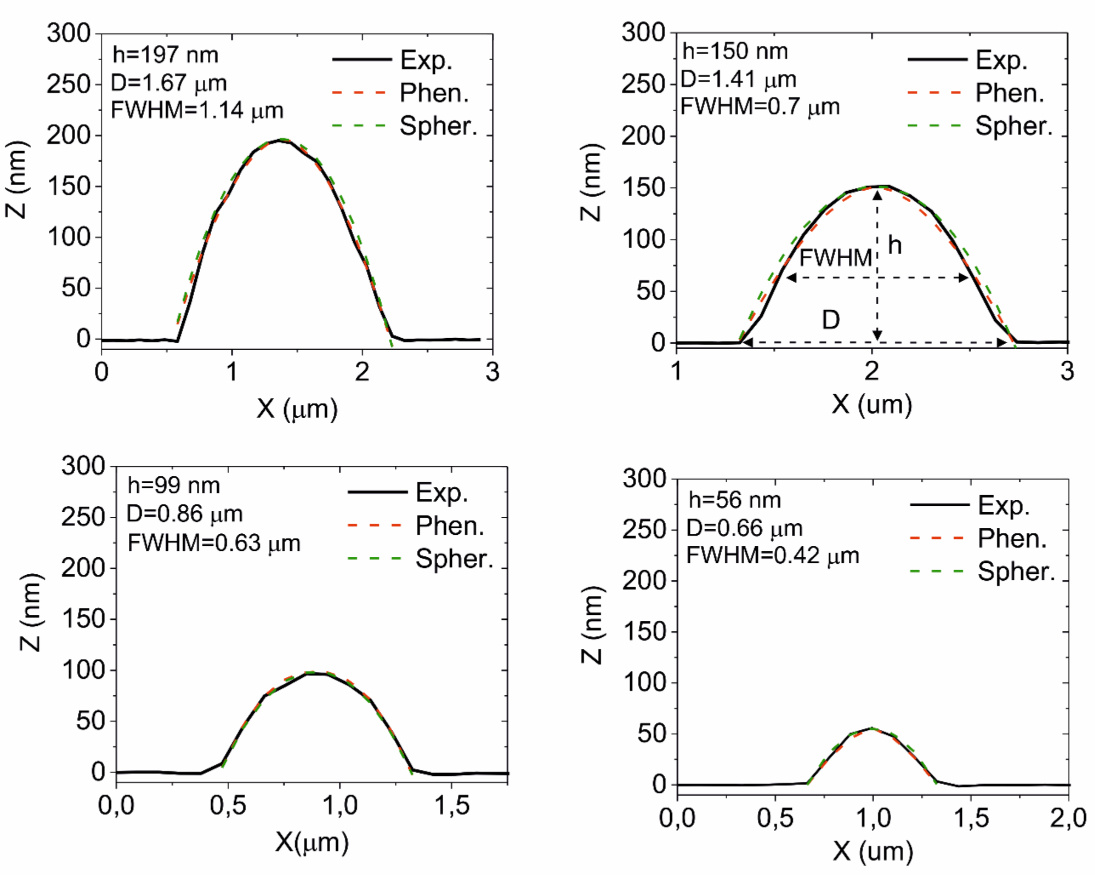

Figure S7: Comparison between the measured topography of four adsorbed liposomes (continuous black lines) and the modeled geometries according to the phenomenological function in Eq. (S12) (red dahsed lines) and the spherical cap function in Eq. (S13) (green dashed lines).

We note that for the DOPC liposomes adsorbed on the substrate, we have found linear relationships between the parameters *h*, *FWHM* and *D*, namely

$$h(nm) = -39.9 + 0.14D(nm)$$
$$FWHM\,(nm) = -99.8 + 0.75D(nm) \qquad \text{(S15)}$$

The relationships in Eq. (S15) together with Eq. (S12) allows generating liposome geometries of any size compatible with the shape of the adsorbed liposomes. We used this fact, for instance, to generate generic theoretical predictions, as those in Fig. 3 of the main manuscript.

References

1. Polyanin, A. D.; Manzhirov, A. V. *Handbook of Mathematics for Engineers and Scientists;* Champam&Hall/CRC Press: Boca Raton, 2006; p 69.

2. Jones, T. B. *Electromechanics of particles;* Cambridge University Press: Cambridge, 1995.

3. Giordano, S. Dielectric and Elastic Characterization of Nonlinear Heterogeneous Materials. *Materials* **2009,** *2,* 1417-1479.
